# Human Bocavirus NS1 and NS1-70 Proteins Inhibit TNF-α-Mediated Activation of NF-κB by targeting p65

**DOI:** 10.1038/srep28481

**Published:** 2016-06-22

**Authors:** Qingshi Liu, Zhenfeng Zhang, Zhenhua Zheng, Caishang Zheng, Yan Liu, Qinxue Hu, Xianliang Ke, Hanzhong Wang

**Affiliations:** 1 Key Laboratory of Special Pathogens and Biosafety, Center for Emerging Infectious Diseases, Wuhan Institute of Virology, Chinese Academy of Sciences, Wuhan 430071, China; 2 University of Chinese Academy of Sciences, Beijing 100049, China; 3 State Key Laboratory of Virology, Wuhan Institute of Virology, Chinese Academy of Sciences, Wuhan 430071, China

## Abstract

Human bocavirus (HBoV), a parvovirus, is a single-stranded DNA etiologic agent causing lower respiratory tract infections in young children worldwide. Nuclear factor kappa B (NF-κB) transcription factors play crucial roles in clearance of invading viruses through activation of many physiological processes. Previous investigation showed that HBoV infection could significantly upregulate the level of TNF-α which is a strong NF-κB stimulator. Here we investigated whether HBoV proteins modulate TNF-α–mediated activation of the NF-κB signaling pathway. We showed that HBoV NS1 and NS1-70 proteins blocked NF-κB activation in response to TNF-α. Overexpression of TNF receptor-associated factor 2 (TRAF2)-, IκB kinase alpha (IKKα)-, IκB kinase beta (IKKβ)-, constitutively active mutant of IKKβ (IKKβ SS/EE)-, or p65-induced NF-κB activation was inhibited by NS1 and NS1-70. Furthermore, NS1 and NS1-70 didn’t interfere with TNF-α-mediated IκBα phosphorylation and degradation, nor p65 nuclear translocation. Coimmunoprecipitation assays confirmed the interaction of both NS1 and NS1-70 with p65. Of note, NS1 but not NS1-70 inhibited TNF-α-mediated p65 phosphorylation at ser536. Our findings together indicate that HBoV NS1 and NS1-70 inhibit NF-κB activation. This is the first time that HBoV has been shown to inhibit NF-κB activation, revealing a potential immune-evasion mechanism that is likely important for HBoV pathogenesis.

TNF-α and cell products induced by viral and bacterial infection (e.g., IL-1, dsRNA, LPS) or cellular stresses (e.g., phorbol esters, UV) activate the NF-κB signaling pathway^1^,^2^. NF-κB acts broadly to influence gene expression, which affects cell survival, differentiation, and proliferation regardless of its most important and evolutionarily conserved role in the immune system^3^. TNF-α is a proinflammatory cytokine significantly affecting the regulation of inflammatory responses as well as cell-cycle proliferation and apoptosis^4^. TNF-α exerts its function as a trimer by binding to either TNF-R1 or TNF-R2^5^. TNF-R1 then recruits the adaptor protein TNFR-associated death domain (TRADD) through the death domain (DD) interaction, subsequently recruiting TRAF2. A signaling cascade culminating in the activation of IκB kinase (IKK) is initiated by these adaptor signaling proteins. The IKK complex consists of two catalytic subunits IKKα and IKKβ and a regulatory subunit IKKγ. IKK phosphorylates the inhibitory IκBα subunit of the NF-κB·IκBα complex in the cytoplasm. Phosphorylation of IκBα leads to ubiquitination, targeting IκBα for degradation by the proteasome and then releasing NF-κB from the inhibitory complex. The freed NF-κB (p50/p65 heterocomplex) proteins are transported into the nucleus, where they bind to their target sequences and activate gene transcription^3^,^5^,^6^.

NF-κB, particularly the p65 subunit, undergoes various post-translational modifications, including ubiquitination, phosphorylation, acetylation, SUMOylation, nitrosylation, and methylation. These modifications play a key role in determining the duration and strength of NF-κB nuclear activation, as well as its transcriptional output^7^,^8^,^9^. Protein p65 can be phosphorylated both in the cytoplasm and nucleus in response to various stimuli, whose phosphorylation sites are mainly within the N-terminal Rel homology domain (RHR) and the C-terminal transcriptional activation domain (TAD). Serine 536 of p65 is targeted for phosphorylation under various conditions by different kinases, including IKKs, ribosomal subunit kinase-01 (RSK1), and TANK binding kinase (TBK1) with different functional consequences^7^,^9^. For instance, phosphorylation of p65 at Ser-536 by IKKβ induced by TNF-α increases p300 binding, thus enhancing p65 acetylation at Lys-310 and enhancing the overall transcriptional activity of NF-κB^10^.

Human bocavirus (HBoV) belongs to the genus *Bocaparvovirus* of the Parvoviridae family^11^,^12^. HBoV genome, which is approximately 5.5 kb in length, encodes two structural proteins (VP1 and VP2) and three nonstructural proteins (NS1, NS1-70 and NP1)^13^,^14^. During the submission of this article, novel small (NS) proteins (NS2, NS3, and NS4) have been identified^15^. HBoV often coinfects hosts with other respiratory viruses and causes lower respiratory tract diseases^16^,^17^,^18^,^19^. Severe and deadly cases associated with high viral load, anti-HBoV IgM antibody detection, or increased IgG antibody production have been documented^17^,^20^,^21^,^22^.

To circumvent the innate immune responses, different viruses have developed various strategies^23^,^24^,^25^. We previously reported that the HBoV NP1 protein blocks IRF3 binding to the IFNB promoter by interacting with the DNA-binding domain of IRF-3, resulting in downregulation of IFN-β production^26^. The HBoV VP2 protein inhibits proteasome-dependent degradation of RIG-I by interacting with RNF125, a negative regulator of the IFN pathway, resulting in upregulation of IFN-β^27^. Investigation with clinical samples showed that HBoV infection could significantly upregulate the level of TNF-α^28^. Nevertheless, it is remains unclear whether HBoV has evolved strategies to interfere with TNF-α-induced NF-κB activation in order to evade the immune responses of the host. The current study demonstrated that the nearly full-length HBoV clone inhibited TNF-α-induced NF-κB activation. We also examined the role of HBoV NS1 and NS1-70 proteins in the modulation of host innate immunity. NS1 and NS1-70 interacted with the DNA-binding domain of p65 and blocked its association with the κB DNA element. NS1 suppressed p65 phosphorylation, whereas NS1-70 failed to suppress p65 phosphorylation. Our findings together imply that inhibition of NF-κB by NS1 and NS1-70 may facilitate the replication and pathogenesis of HBoV and concurrent pathogens.

## Results

### Nearly full-length clone of HBoV inhibits TNF-α-induced NF-κB activation

**Figure 1:**
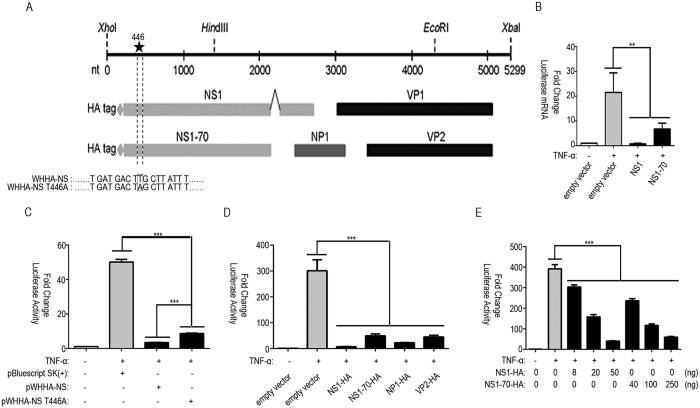
pWHHA-NS, NS1, and NS1-70 inhibit TNF-α-induced NF-κB activation. (**A**) Schematic of the HBoV genome and strategy for constructing pWHHA-NS and pWHHA-NS T446A. ORFs are shown in the lower panel. Sites for nearly full-length cloning are shown at the top of the panel. The HA tag at the N termini of NS1 and NS1-70 is denoted by a “diamond” symbol. Codons are shown as three-letter segments. T446A mutation is framed and denoted by a “star” symbol. (**B**) Luciferase mRNA analysis. The 293T cells in 6-well plates were cotransfected with 0.5 μg of pNF-κB-luc, 0.1 μg of pRL-TK, and 2 μg of NS1, NS1-70 plasmid or empty vector for 24 h and then mock-treated or treated with TNF-α (10 ng/ml) for 6 h. RT-PCR was performed using luciferase and Renilla primers. The resultant ratios were normalized to the fold-change value by that of TNF-α-untreated cells transfected with the empty vector, pNF-κB-luc and pRL-TK. Data shown represent three independent experiments, with each determination performed in duplicate (mean ± SD of fold change). Asterisks indicate significant differences between groups (**p < 0.05, Student’s t-test). (**C–E**) 293T cells in 24-well plates were cotransfected with 125 ng pNF-κB-luc, 25 ng pRL-TK and 0.5 μg pWHHA-NS, 150 ng pWHHA-NS T446A (**C**), 0.5 μg HBoV gene expression plasmid (**D**), indicated amount of NS1 and NS1-70 expression plasmid (**E**), or empty vector for 24 h. Cells were then mock-treated or treated with TNF-α (10 ng/ml) for 6 h. Reporter activity was determined by dual-luciferase reporter assays. The resultant ratios were normalized to the fold-change value by that of TNF-α-untreated cells cotransfected with empty vector, pNF-κB-luc and pRL-TK. Data shown represent three independent experiments, with each determination performed in duplicate (mean ± SD of fold change). Asterisks indicate significant differences between groups (***p < 0.01, Student’s t-test).

TNF-α plays an important role in host defense against viral infection. In HBoV infection, the elevated level of TNF-α is detected in serum^28^. We evaluated the inhibitory activity of the HBoV nearly full-length clone (pWHHA-NS) containing the HBoV coding sequence (nt 1–5299) (Fig. 1A) on NF-κB activation by using an NF-κB promoter reporter system. Reporter plasmid pNF-κB-luc and internal control plasmid pRL-TK, together with pWHHA-NS or empty vector, were cotransfected into 293T cells. At 24 h post-transfection, cells were either mock-treated or treated with human TNF-α (10 ng/ml) for 06 h. The nearly full-length HBoV clone significantly inhibited TNF-α stimulated NF-κB promoter activity (Fig. 1C).

### HBoV NS1 and NS1-70 inhibit TNF-α-induced NF-κB activation

To determine viral protein(s) that contribute to the NF-κB inhibitory activity of the nearly full-length HBoV clone, we constructed mammalian expression vectors encoding different HBoV proteins. We evaluated the effect of each construct on the modulation of NF-κB production by using the NF-κB promoter reporter system. NS1 exhibited severely strong inhibition on NF-κB promoter activity with an inhibition rate of 98%. NS1-70, NP1 and VP2 also showed significant inhibition on NF-κB promoter activity with an inhibition rate of 84% for NS1-70, 93% for NP1 and 85% for VP2 (Fig. 1D).

Further we focused on NS1 and its N terminal isoform NS1-70. We first examined the effect of NS1 and NS1-70 on the inhibition of NF-κB activation. With the increase in the concentration of NS1 or NS1-70 expressing plasmid, NF-κB activation was suppressed in a dose-dependent manner (Fig. 1E). Real-time PCR was subsequently performed to determine whether NS1 and NS1-70 affected NF-κB activation at the mRNA level. While TNF-α stimulation upregulated the level of luciferase mRNA, NS1 and NS1-70 strongly suppressed the upregulation induced by TNF-α. Without TNF-α stimulation, the level of luciferase mRNA was hardly detectable (Fig. 1B).

Then to determine whether NS1 and NS1-70 accounted for the inhibitory activity of the nearly full-length HBoV clone (pWHHA-NS) on NF-κB activation, we constructed an NS1 and NS1-70-deleted nearly full-length HBoV clone (pWHHA-NS T446A). We subsequently performed an NF-κB promoter reporter assay to verify the role of NS1 and NS1-70. The inhibitory activity of pWHHA-NS T446A was much weaker than that of pWHHA-NS in response to TNF-α stimulation, indicating that NS1 and NS1-70 contributed to the efficient inhibition of NF-κB activation (Fig. 1C). These results indicate that NS1 and NS1-70 inhibit TNF-α-induced NF-κB transcriptional activation.

### HBoV NS1 and NS1-70 interfere with NF-κB pathway

**Figure 2:**
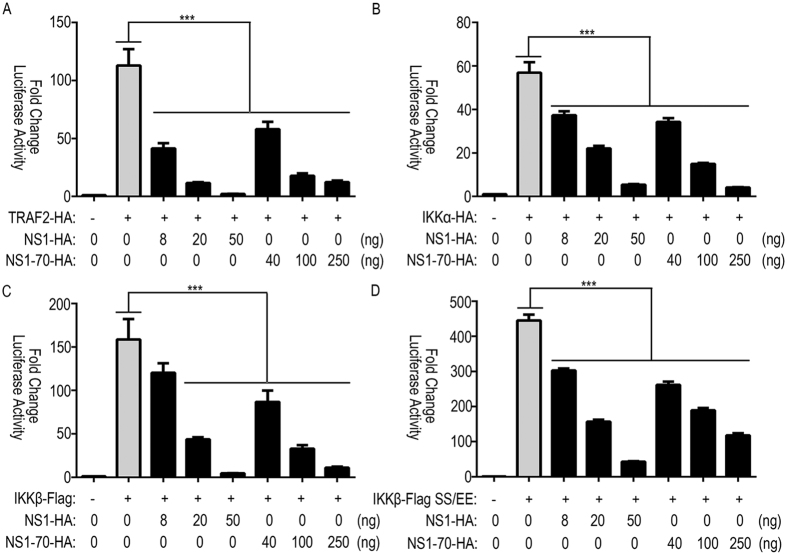
Effect of NS1 and NS1-70 on the NF-κB pathway. 293T cells in 24-well plates were cotransfected with 125 ng of pNF-κB-luc, 25 ng of pRL-TK and either HA-TRAF2 (**A**), HA-IKKα (**B**), FLAG-IKKβ (**C**), or FLAG-IKKβ SS/EE (**D**) together with the indicated amounts of NS1 and NS1-70 expression plasmids. Total amounts of transfected DNA were kept equal by adding empty vector. Reporter activity was determined 30 h post-transfection by the dual-luciferase reporter assays. The resultant ratios were normalized to the fold-change value by that of cells cotransfected with empty vectors, pNF-κB-luc and pRL-TK. Data represent at least 3 independent experiments, with each determination performed in duplicate (mean ± SD of fold-change). Asterisks indicate significant differences between groups (***p < 0.01, Student’s t-test).

To further elucidatethe inhibitory mechanism of NS1 and NS1-70 on NF-κB activation, we performed experiments to determine how NS1 and NS1-70 proteins interfere with the TNF-α-mediated NF-κB activation pathway. We examined the effects of NS1 and NS1-70 proteins on luciferase activity mediated by overexpression of TNF-α signaling transducers, including TRAF2, IKKα, IKKβ, and IKKβ SS/EE. NS1 and NS1-70 inhibited TRAF2-induced NF-κB activation in a dose-dependent manner: transfection of 8, 20, or 50 ng of NS1 or 40, 100, or 250 ng of NS1-70-expressing plasmid significantly suppressed the 100 ng TRAF2 mediated-luciferase activity (63%, 89%, and 98% or 48%, 84%, and 89% reduction, respectively) (Fig. 2A). Similar to the results described above, NS1 and NS1-70 expression reduced 100 ng of IKKα, 100 ng of IKKβ and 50 ng of IKKβ SS/EE-mediated luciferase activity in a dose dependent-manner (decreases ranging from 34–90%, 24–97%, and 32–90%, as well as 40–93%, 45–93%, and 41–74% decreases, respectively) (Fig. 2B–D).

### IκBα phosphorylation and degradation are not suppressed by HBoV NS1 and NS1-70

**Figure 3:**
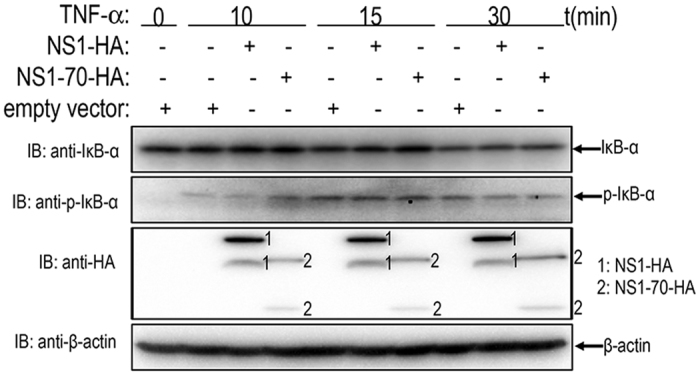
Examination of TNF-α stimulated IκBα degradation and phosporylation in NS1 and NS1-70-expressing cells. 293T cells transfected with empty vector, NS1 or NS1-70-expressing plasmid were stimulated with TNF-α (20 ng/ml) for indicated durations. Equal amounts of cell lysates were analyzed by immunoblotting with the anti-IκBα antibody or the anti-phospho-IκBα antibody. The results represent three independent experiments. The protein levels of NS1 and NS1-70 as well as β-actin in the same cell lysates were determined by immunoblotting.

Activated phospho-IKKβ can phosphorylate IκBα and cause subsequent degradation. To address whether NS1 and NS1-70 inhibit NF-κB activation by preventing phosphorylation and degradation of IκBα, we examined the phosphorylation and degradation of IκBα by immunoblotting. When treated with TNF-α, the amount of IκBα was equally decreased in both NS1 and NS1-70-expressing and empty vector-transfected cells (Fig. 3). This reduction indicated that TNF-α-induced IκBα degradation was not inhibited by NS1 and NS1-70. Phosphor-IκBα was also detected when cells were treated with TNF-α for 10, 15, or 30 min. The level of phosphor-IκBα in empty vector-transfected cells was indistinguishable to that in NS1 and NS1-70-expressing cells at the indicated time (Fig. 3). These results demonstrate that NS1 and NS1-70 proteins do not interfere with TNF-α-mediated IκBα phosphorylation and degradation.

### HBoV NS1 and NS1-70 do not suppress p65 nuclear translocation

**Figure 4:**
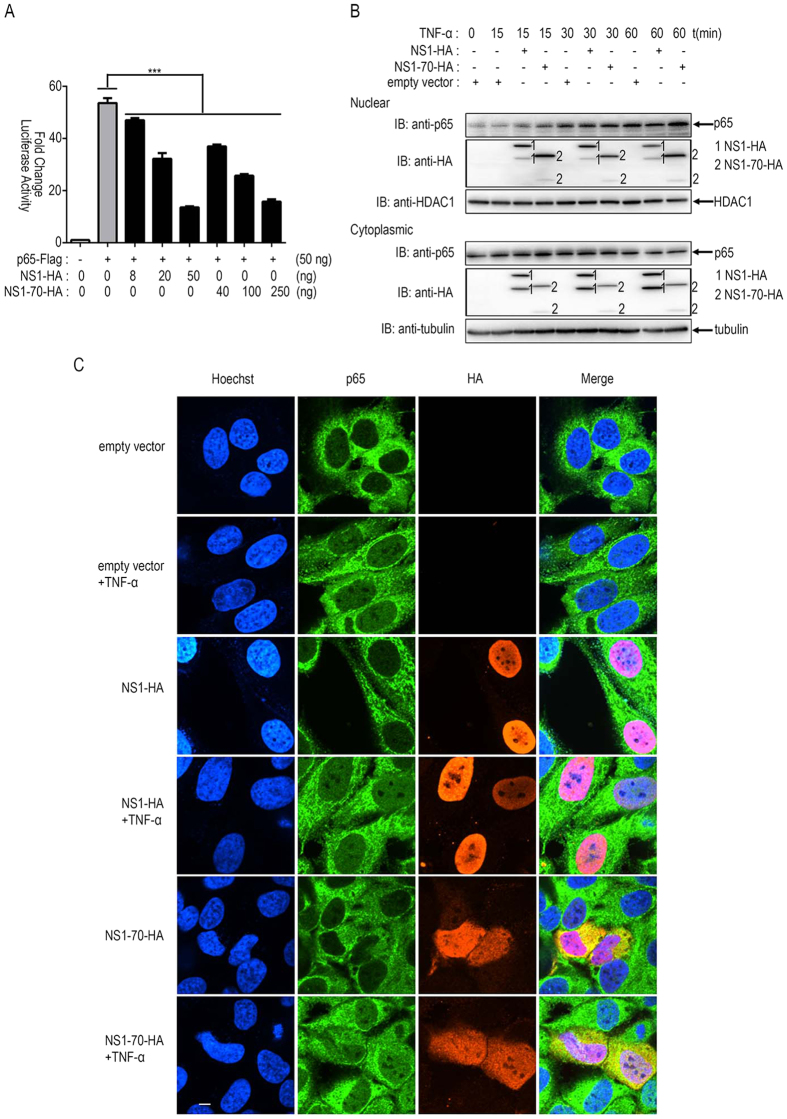
HBoV NS1 and NS1-70 do not block p65 nuclear translocation. (**A**) 293T cells in 24-well plates were cotransfected with 125 ng of pNF-κB-luc, 25 ng of pRL-TK, and FLAG-p65 together with specific amounts of NS1 or NS1-70 expression plasmids. Total amounts of transfected DNA were kept equal by adding an empty vector. Reporter activity was determined 30 h post-transfection by dual-luciferase reporter assays. The resultant ratios were normalized to the fold-change values by that of TNF-α-untreated cells cotransfected with empty vectors, pNF-κB-luc and pRL-TK. Data represent at least 3 independent experiments, with each determination performed in duplicate (mean ± SD of fold-change). Asterisks indicate significant differences between groups (***p < 0.01, Student’s t-test). (**B**) 293T cells were transfected with NS1, NS1-70 expression plasmid, or empty vector for 30 h and then mock-treated or treated with TNF-α (10 ng/ml) for 15, 30, or 60 min. Equal amounts of cytoplasmic and nuclear extracts were resolved by immunoblotting with the anti-p65 antibody or the anti-HA antibody. HDAC1 and tubulin were used as loading controls for nuclear and cytoplasmic proteins, respectively. (**C**) HeLa cells were transfected with NS1, NS1-70 expression plasmid, or empty vector for 30 h. The cells were then mock-treated or treated with TNF-α (10 ng/ml) for 30 min. HeLa cells were subjected to immunofluorescence staining for detection of p65 subcellular localization by using rabbit anti–p65 and FITC-conjugated secondary Ab (green). NS1 and NS1-70 expression levels were detected using a mouse anti-HA tag and Texas Red-conjugated secondary Ab (red). Nuclei were stained by Hoechst 33258 (blue). One of three experiments is shown.

Upon stimulation of cytokine receptors such as those in the TNF receptor superfamily, membrane proximal events lead to the activation of the IKK. Phosphorylation of IκBα results in proteasomal degradation and releases NF-κB (p50/p65 heterocomplex) for nuclear translocation and activation of gene transcription^6^. Protein p65 is a key transcription factor. We determined whether NS1 and NS1-70 interfered with these processes. We examined the effects of NS1 and NS1-70 proteins on luciferase activity mediated by overexpression of p65. NS1 and NS1-70 inhibited p65-induced NF-κB activation in a dose-dependent manner: transfection of 8, 20, or 50 ng of NS1 or 40, 100, or 250 ng of NS1-70-expressing plasmid significantly suppressed the 50 ng p65 mediated-luciferase activity (12%, 40%, and 75% or 31%, 52%, and 71% reduction, respectively) (Fig. 4A).

To further test whether NS1 and NS1-70 interfere with the nuclear translocation of p65, 293T cells and HeLa cells were transfected with NS1 or NS1-70 expression plasmid, or empty vector for 30 h. For 293T cells mock-treated or treated with TNF-α for 15, 30, or 60 min, cytoplasmic and nuclear proteins were subsequently prepared, followed by immunoblotting to determine the distribution of p65 (Fig. 4B). For HeLa cells mock-treated or treated with TNF-α for 30min, immunofluorescence assays were performed to examine the location of p65 (Fig. 4C). TNF-α-stimulation induced the nuclear translocation of p65, whereas NS1 and NS1-70 exerted negligible effects on the location of p65 (Fig. 4B,C). This result indicates that NS1 and NS1-70 does not inhibit p65 nuclear translocation. However, the significant colocalization of p65 and NS1 (or NS1-70) in nuclear regions suggests a physical interaction between p65 and NS1 (or NS1-70) (Fig. 4B,C).

### HBoV NS1 and NS1-70 interact with endogenous p65

**Figure 5:**
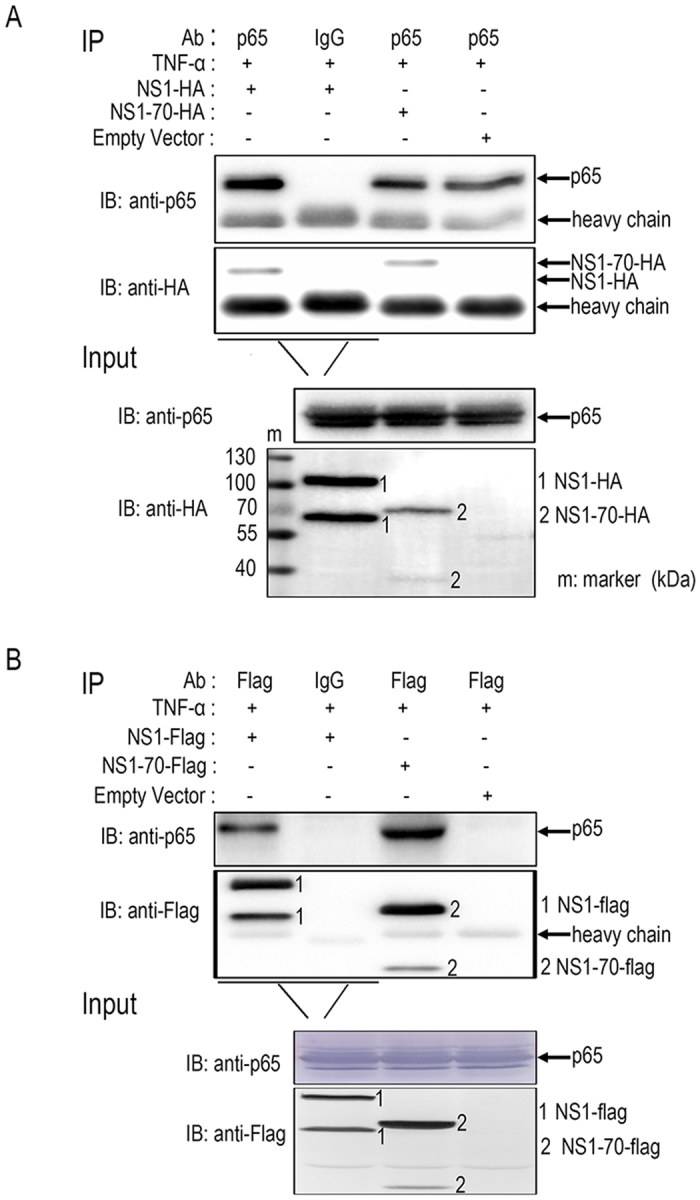
Analysis of the interaction between p65 and NS1 (or NS1-70). (**A,B**) 293T cells were transfected with NS1 (NS1-HA or NS1-FLAG) or NS1-70 (NS1-70-HA or NS1-70-FLAG) expression plasmids or empty vectors for 30 h. The cells were then treated with TNF-α (10 ng/ml) for 30 min. Cells were lysed and subjected to immunoprecipitation (IP) using mouse anti-FLAG tag or rabbit anti-p65. Mouse (or rabbit) IgG was used as negative control. IP products and 5% input samples were resolved by immunoblotting (IB). Rabbit anti–p65 was used for detection of p65. For detection of FLAG-tagged and HA-tagged proteins, the indicated mouse Abs were used. One of three experiments is shown.

NS1 and NS1-70 interfered with the NF-κB pathway but did not inhibit p65 nuclear translocation. Thus, we further investigated the underlying mechanisms. The significant colocalization of p65 with both NS1 and NS1-70 in nuclear regions suggests a physical interaction between p65 and NS1 (or NS1-70) (Fig. 4B,C). NS1 (NS1-HA or NS1-FLAG) or NS1-70 (NS1-70-HA or NS1-70-FLAG) expression plasmids was transfected into 293T cells. 30 h post-transfection, cells were treated with TNF-α for 30 min. Co-IP assays were performed to determine the interaction between NS1 (or NS1-70) and p65 (Fig. 5A,B). NS1-HA (or NS1-70-HA) was immunoprecipitated with endogenous p65 using anti-p65 (Fig. 5A). A similar result was observed in reciprocal Co-IP assays using the anti-FLAG tag (Fig. 5B). These results demonstrate that NS1 and NS1-70 proteins interact with p65.

### HBoV NS1 and NS1-70 block the association of p65 with κB DNA element

**Figure 6:**
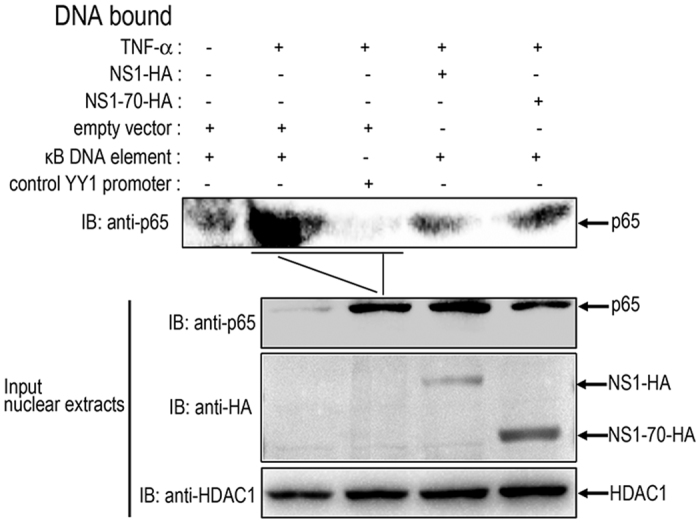
NS1 and NS1-70 block the association of p65 with the κB DNA element. 293T cells were transfected with NS1-HA or NS1-70-HA expression plasmid or empty vectors for 30 h, The cells were mock-treated or treated with TNF-α (10 ng/ml) for 60 min. Nuclear extracts were then isolated and subjected to DNA affinity-binding assay by using Streptavidin magnetic particles and biotinylated dsDNA oligonucleotides containing the κB DNA element sequence or the control oligonucleotide YY1 promoter sequence. DNA-bound proteins and 5% of input nuclear extracts were analyzed by immunoblotting to detect p65 using anti-p65 antibody and NS1-HA together with NS1-70-HA using the anti-HA antibody. HDAC1 was used as a loading control to demonstrate that equal amounts of nuclear proteins were present in various samples. One of three experiments is shown.

Phosphorylation of IκBα leads to its proteasomal degradation and releases NF-κB (p50/p65 heterocomplex) from the inhibitory complex. The freed NF-κB proteins are then transported into the nucleus, where they bind to their target sequences (κB DNA element) and activate gene transcription^3^,^6^. The interaction of NS1 and NS1-70 with p65 probably leads to an interrupted association of p65 with its responsive DNA. A DNA-binding affinity assay using dsDNA oligo containing the κB DNA element was performed to test this hypothesis. 293T cells were transfected with the NS1 or NS1-70 expression plasmid or empty vector for 30 h. The cells were then mock-treated or treated with TNF-α for 60 min. The nuclear fractions were isolated, and equal amounts of nuclear proteins were used for κB DNA element and control oligonucleotide YY1 promoter binding. DNA-bound products were analyzed by immunoblotting to assess the quantity of endogenous p65. Protein p65 accumulated in the nucleus upon TNF-α stimulation and efficiently bound to the κB DNA element in cells transfected with empty vectors (Fig. 6). The oligonucleotide YY1 promoter which was used as a control failed to bind the p65 protein (Fig. 6). In the presence of NS1 and NS1-70, the binding of p65 to the κB DNA element was significantly reduced (Fig. 6), but the amount of p65 accumulation in the nucleus was not affected. This result indicates that NS1 and NS1-70 can block the association of p65 with the κB DNA element.

### HBoV NS1 and NS1-70 interact with p65 N-terminal domain (NTD)

**Figure 7:**
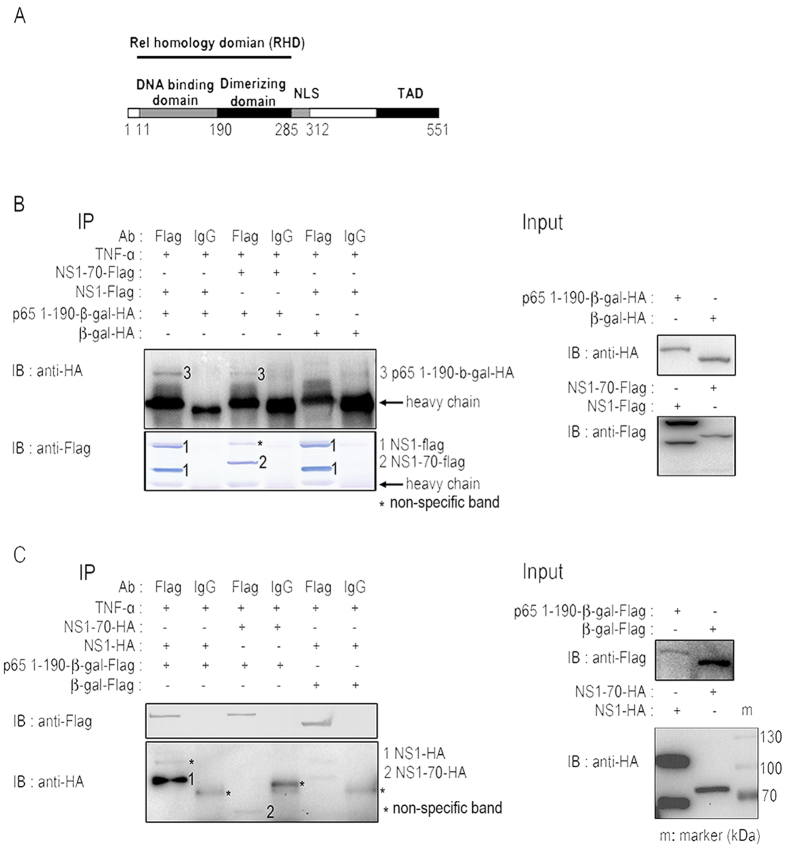
Analysis of the interaction between p65 1-190 and NS1 (or NS1-70). 293T cells were transfected separately with NS1 (NS1-HA or NS1-FLAG) or NS1-70 (NS1-70-HA or NS1-70-FLAG) or either p65 1-190-β-gal (p65 1-190-β-gal -HA or p65 1-190-β-gal-FLAG) or β-gal (β-gal-HA or β-gal-FLAG) expression plasmids for 30 h. All cells were then treated with TNF-α (10 ng/ml) for 30 min. The cells were lysed and subjected to immunoprecipitation (IP) using the mouse anti-FLAG tag. Mouse IgG was used as negative control. IP products and 5% input samples were analyzed by immunoblotting. For detection of FLAG-tagged and HA-tagged proteins, the indicated mouse Abs were used. One of three experiments is shown.

Protein p65 consists of 551 aa, including a Rel homology domain (RHR), a nuclear localization signal (NLS) and a transcriptional activation domain (TAD). The RHR domain of p65 forms into two distinct domains connected by a linker, the N-terminal domain (NTD) and the C-terminal domain (CTD). The NTD recognizes the target DNA sequence through a specific interaction with DNA bases (Fig. 7A)^6^,^9^. We hypothesize that NS1 and NS1-70 interrupt the association of p65 with its responsive DNA by interacting with p65 NTD (1-190 aa). We tested this hypothesis by generating p65 truncations p65 1-190-β-gal-HA and p65 1-190-β-gal-FLAG. NS1 (NS1-HA or NS1-FLAG) or NS1-70 (NS1-70-HA or NS1-70-FLAG) or either p65 1-190-β-gal (p65 1-190-β-gal-HA or p65 1-190-β-gal-FLAG) or β-gal (β-gal-HA or β-gal-FLAG) expression plasmids were transfected separately into 293T cells (Fig. 7B,C). All cells were treated with TNF-α for 30 min at 30 h post-transfection. Co-IP assays were performed to determine the interaction between NS1 (or NS1-70) and p65 1-190. Protein p65 1-190-β-gal-HA was immunoprecipitated with NS1 (and NS1-70) by using anti-FLAG tag Ab (Fig. 7B). Similar results were observed in reciprocal Co-IP assays using anti-FLAG tag (Fig. 7C). These results demonstrate that NS1 and NS1-70 proteins interact with p65 NTD.

### HBoV NS1 but not NS1-70 suppresses p65 phosphorylation

**Figure 8:**
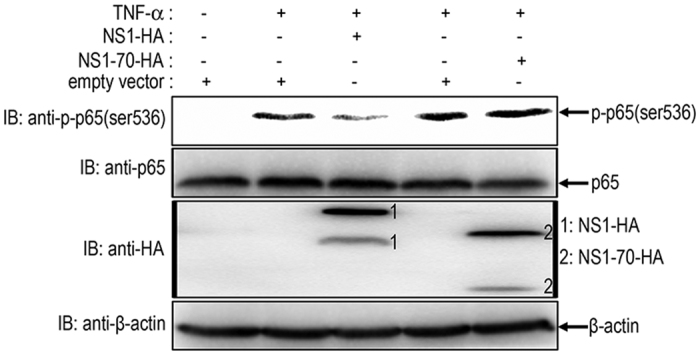
HBoV NS1, rather than NS1-70, suppresses p65 phosphorylation. 293T cells were transfected with NS1 or NS1-70 expression plasmid or empty vector for 30 h. The cells were then mock-treated or treated with TNF-α (20 ng/ml) for 15 min. Equal amounts of cell lysates were analyzed by immunoblotting with anti-p-p65 (Ser536) antibody or anti–p65 antibody. The results represent three independent experiments. The protein levels of NS1-HA, NS1-70-HA, and β-actin in the same cell lysates were determined by immunoblotting.

Numerous regulatory post-translational modifications (PTMs) of NF-κB have been reported to affect transcriptional responses. The p65 subunit also undergoes multiple modifications such as phosphorylation^3^,^9^. Both NS1 and NS1-70 interact with p65 and block the association of p65 with the κB DNA element. However, NS1 appeared to play a stronger role in interfering with the NF-κB pathway than did NS1-70 (Figs 1E, 2 and 4A). To address the underlying mechanisms, NS1 or NS1-70 expression plasmid or empty vector was transfected into 293T cells. 30 h post-transfection, cells were mock-treated or treated with TNF-α for 15 min. TNF-α stimulation induced p65 phosphorylation at ser536 (Fig. 8). The level of phosphor-p65 in NS1-expressing cells was lower than that in vector-transfected cells (Fig. 8). Overexpression of NS1-70 did not inhibit p65 phosphorylation at ser536 (Fig. 8). These results demonstrate that NS1 rather than NS1-70 protein inhibits TNF-α-mediated p65 phosphorylation at ser536.

## Discussion

Human bocavirus (HBoV) DNA is frequently detected in the airways of young children with respiratory symptoms^16^,^29^; however, its etiologic role remains controversial. A reverse genetics system has recently been established to study the molecular biology and pathogenesis of HBoV by using an *in vitro* culture model of HAE. HBoV-infected HAE either at low or high multiplicity of infection shows signs of lung airway-tract injury, including the disruption of the tight junction barrier, loss of cilia, and epithelial cell hypertrophy. The occurrence of these symptoms indicates that HBoV is a viral pathogen^13^,^30^,^31^. Given that interactions between viruses and host innate immune responses are crucial for viral replication, in the present study, we focused on the modulation of innate immunity by a nearly full-length HBoV clone and HBoV-encoded proteins. We found that the nearly full-length HBoV clone significantly inhibited NF-κB. We subsequently identified the nonstructural proteins NS1 and NS1-70 as antagonists of NF-κB. Both NS1 and NS1-70 suppressed NF-κB by binding to the DNA-binding domain of p65 upon stimulation of TNF-α. Such suppression blocked the association between NF-κB and the κB DNA element. Interestingly, NS1, rather than NS1-70, inhibited the phosphorylation of p65. Our study reveals a potential mechanism by which HBoV evades human innate immune responses.

The interaction between HBoV and the NF-κB pathway has not been characterized previously. Evidences from clinical studies indicate that HBoV increases the serum concentrations of TNF-α (the activator of NF-κB), IL-6, and IL-8 (regulated by NF-κB)^28^,^32^. Considering that the NF-κB pathway is an attractive target for common human viral pathogens^33^, we asked whether HBoV has evolved strategies to interfere with the NF-κB pathway to evade the immune responses of the host. Although primary human airway epithelial (HAE) cell culture system has been shown to support HBoV1 infection, the use of this approach is limited by the variability between donors, tissue availability. In the current study, we used a nearly full-length HBoV clone containing entire coding sequences. The gene-expression profile of the nearly full-length clone was similar to that of HBoV with all open reading frames under control of their native promoters. Our study indicated that the nearly full-length HBoV clone and the nonstructural proteins NS1 and NS1-70 interfered with NF-κB upon stimulation with TNF-α. In addition, NS1 and NS1-70 proteins interfered with TNF-α signaling transducers, including TRAF2, IKKα, IKKβ, and IKKβ SS/EE, suggesting that NS1 and NS1-70 are essential for viral DNA replication and likely function as NF-κB antagonists to evade innate immunity during HBoV infection.

Activation of NF-κB rapidly occurs within minutes from stimulation. The process requires no protein synthesis and can influence critical steps in the host cell life, making the NF-κB pathway an attractive target for invading viruses^33^. Human pathogens such as HIV-1, the human T-cell leukemia virus, and hepatitis B viruses have developed different strategies to modulate the NF-κB pathway and particularly IKK activation^33^,^34^. However, few viral proteins have been reported to inhibit the activation of NF-κB DNA binding. For example, the African swine fever virus A238L protein acts within the nucleus to inhibit the binding of NF-κB to κB enhancer sequences^35^. In the current study, overexpression of HBoV NS1 and NS1-70 affected neither the phosphorylation, ubiquitination, proteasomal degradation of IkBα nor the level of p65 expression and nuclear translocation. However, NS1 and NS1-70 significantly blocked the association of p65 with the κB DNA element by interacting with the DNA-binding domain of p65. Interaction of NS1 and NS1-70 with the p65 DNA-binding domain probably disables the access of p65 to its responsive elements in the target genes, thereby interrupting its target gene transcription.

The final step in NF-κB signaling that can be modulated is transactivation of target genes^34^. Following removal of IkBα from the NF-κB complex, NF-κB can accumulate in the nucleus and bind to the κB DNA element in promoters and enhancers of target genes. However, NF-κB transcriptional response activity is affected by multiple transcriptional coregulators, including both histone acetyltransferases, including CBP and p300 and numerous regulatory PTMs of p65^3^. Several viruses or viral proteins are known to modulate the final step. For example, the Epstein–Barr virus immediate–early BZLF1 protein induces accumulation of nuclear NF-κB, however, inhibits NF-κB-dependent gene expression by reducing the ability of NF-κB to bind to NF-κB responsive promoters in the context of the intact cellular genome^36^. To our knowledge, a viral protein capable of inhibiting PTMs of p65 has not been reported previously. We identified that NS1 inhibited TNF-α-mediated p65 phosphorylation at ser536. Interestingly, NS1-70, which is a relative small isoform of NS1 lacking the C-terminus, showed no inhibitory activity on p65 phosphorylation at ser536. This may provide a molecular explanation why NS1 appeared to be more potent in interfering with the NF-κB pathway than did NS1-70. As described above, upon stimulation by TNF-α, phosphorylation of p65 at Ser-536 by IKKβ increases p300 binding, thereby enhancing p65 acetylation at Lys-310 and the overall transcriptional activity of NF-κB^10^. However, p300 cannot relieve the inhibition activity of NS1 and NS1-70 (data not shown). Although both NS1 and NS1-70 appear to interact with p65, NS1 likely inhibits p65 phosphorylation at ser536 through its C-terminus structure by masking p65 TAD and consequently preventing the access of IKKβ. The mechanism by which NS1 inhibits p65 phosphorylation at ser536 requires further investigation.

To avoid the innate immune responses, HBoV may have evolved various strategies. We previous showed that the HBoV NP1 protein blocks IRF3 from binding to the IFNB promoter by interacting with the DNA-binding domain of IRF-3. This process leads to low-level production of IFNs^26^. Through the same process, the HBoV VP2 protein inhibits proteasome-dependent degradation of RIG-I by interacting with RNF125, a negative regulator of the IFN pathway, resulting in high-level production of IFNs^27^. In the current study, we found that all HBoV-encoded proteins including VP2 significantly inhibited TNF-α-induced NF-κB promoter activity. In favor of infection, HBoV which only encodes a few proteins has likely developed different strategies to interfere with different signal pathways.

In conclusion, we demonstrate that HBoV nonstructural proteins NS1 and NS1-70 suppress the NF-κB signal pathway by binding to the p65 DNA-binding domain upon stimulation by TNF-α. Such interaction blocks the association between NF-κB and the κB DNA element, while NS1 but not NS1-70 inhibits the phosphorylation of p65. Our study reveals a potential mechanism by which HBoV evades innate immune responses, providing basis for elucidating HBoV pathogenesis.

## Materials and Methods

### Cells, plasmids, antibodies, and cytokines

The human embryonic kidney 293T cell line and HeLa cell line (both from American Type Culture Collection) were cultured in DMEM (Life Technologies) supplemented with 10% (vol/vol) heat-inactivated FBS (Gibco) at 37 °C with 5% CO_2_.

The full-length HBoV NS1, NS1-70, VP1, VP2, and NP1 genes were generated by polymerase chain reaction (PCR), as described in a previous study^26^. The HBoV (isolate WH, GenBank accession number FJ496754.1) genome was isolated from HBoV-positive stool samples. pWHHA-NS was also described in an earlier study^26^. pFLAG (or HA)-NS1 and pFLAG (or HA)-NS1-70 were constructed by inserting a coding sequence of FLAG (or HA) tag to the C-terminus of NS1 and NS1-70 in the pCAGGS eukaryotic expression vector. pWHHA-NS T446A was constructed by mutating T at nt 446 to A in pWHHA-NS. Plasmid pHA-TRAF2 was provided by Dr. Ashwell (National Institutes of Health, USA)^37^. Plasmids pFLAG-IKKβ and pFLAG-IKKβ SS/EE were provided by Dr. Rao Anjana (Immune Disease Institute, Harvard Medical School, USA)^38^. Plasmid pHA-IKKα was supplied by Dr. Gangmin Hur (Chungnam National University, South Korea)^39^. pNF-κB-luc was purchased from Stratagene, and pRL-TK was purchased from Promega. Plasmid FLAG-p65 was provided by Dr. Jose Alcami (Instituto de Salud Carlos III, Spain)^40^. The coding sequence of β-galactosidase (β-gal) was amplified by PCR using pSV-b-galactosidase control vector (Promega) as the template, and subsequently cloned into the BamHI and HindIII sites of the pCMV-Tag 2B vector. Protein p65 truncation p65 1-190 (residues 1-190) was constructed by PCR and cloned into the pCMV-Tag 2B-FLAG (or HA)-β-gal vector. All constructs were verified by DNA sequencing.

Mouse anti-HA was purchased from Ab-mart (Shanghai, China), and anti-FLAG mAb was obtained from Sigma-Aldrich. Rabbit polyclonal anti-IκBα, p65, and mouse anti-β-actin antibodies were acquired from Proteintech (Wuhan, China). Mouse anti-phospho-IκBα and rabbit anti-phospho-p65 (ser536) antibodies were purchased from Cell Signaling Technology. Rabbit anti-HDAC1 antibody was procured from Beyotime Institute of Biotechnology (China). Rabbit anti-α-Tubulin antibody was from Anbo (Changzhou, China). Rabbit and mouse control IgGs were purchased from Santa Cruz Biotechnology. Human TNF-α was purchased from Prospec (East Brunswick).

### Luciferase reporter assays

In assays involving TNF-α-mediated NF-κB activation, 293T cells were seeded into 24-well plates and cotransfected with 125 ng reporter plasmid pNF-κB-luc, 25 ng Renilla luciferase expression plasmid pRL-TK, and the indicated expression plasmids using ProFection Mammalian Transfection System (Promega). At 24 h post-transfection, cells were mock-treated or treated with TNF-α (10 ng/ml) for 6 h, lysed in a passive lysis buffer (Promega, Madison, USA), and assayed for firefly and Renilla luciferase activities by using the dual luciferase reporter system (Promega, Madison, USA). NF-κB can also be activated by overexpression of TRAF2, IKKα, IKKβ, IKKβ SS/EE, and p65 proteins. Briefly, 293T cells were cotransfected with plasmids expressing TRAF2, IKKα, IKKβ, IKKβ SS/EE, or p65, along with pNF-κB-luc (125 ng), pRL-TK (25 ng), and empty vector pCAGGS or the indicated amount of HBoV protein expression plasmids. In some cases, large amounts of pCAGGS were included in the transfection mixture to equalize the quantity of DNA. At 30 h post-transfection, cells were lysed, and the activities of firefly and Renilla luciferases were measured.

For all assays, experiments were performed in triplicate. For each experiment, firefly luciferase activity was divided by Renilla luciferase activity to correct for differences in transfection efficiencies. The resultant ratios were normalized to the fold-change value obtained from TNF-α-untreated cells cotransfected with pCAGGS or cells cotransfected with pCAGGS, pNF-κB-luc, and pRL-TK.

### Immunoblot and coimmunoprecipitation assays

Transfected cells were lysed in a cell lysis buffer (Beyotime) for immunoblotting. Protease inhibitor cocktail and a phosphatase inhibitor (Roche) were separately added to the lysis buffer to inhibit the degradation and dephosphorylation of the target protein. The supernates of cell lysis were obtained by centrifugation at 16,000 × g for 5 min at 4 °C and subjected to immunoblotting. Immunoblotting analysis was performed as described in a previous study^41^.

For the fractionation of lysates, 293T cells were transfected with NS1-HA, NS1-70-HA, or empty vector for 30 h. The transfected cells were left untreated or treated with 10 ng/ml of TNF-α for 15, 30, or 60 min. Cells were washed, and cytoplasmic and nuclear fractions were isolated using Cytoplasmic/Nuclear protein extraction kit. Protein content was determined using the BCA Protein Assay Kit (both from Beyotime Institute of Biotechnology, China). The fractions were processed as described above for immunoblotting. Tubulin antibody was used as a loading control for cytoplasmic fractions, and HDAC-1 was used as a control for nuclear fractions.

For coimmunoprecipitation experiments, 293T cells were transfected with indicated plasmids. At 30 h post-transfection, cells were treated with 10 ng/ml of TNF-α for 30 min. Cells were harvested and lysed with cell lysis buffer for immunoblotting analysis and immunoprecipitation. Coimmunoprecipitation assay was performed using Dynabeads protein G Immunoprecipitation Kit (Invitrogen). The supernates were incubated with anti-FLAG Ab, anti-p65 Ab, mouse IgG Ab, or rabbit IgG Ab overnight at 4 °C, as indicated. Immunocomplexes were precipitated with 50 μL of Dynabeads Protein G for 2 h at 4 °C. Following several washes with PBS, immunoprecipitates were eluted by boiling in Laemmli sample buffer and analyzed by immunoblotting.

### Immunofluorescence analysis

For colocalization assay, HeLa cells grown on glass slides were transfected with indicated plasmids. At 30 h post-transfection, transfected cells were treated with 10 ng/ml of TNF-α for 30 min, fixed with 4% paraformaldehyde for 10 min and permeabilized with 0.2% Triton X-100 for 15 min. Microscopy analysis was performed as described in a previous study^26^.

### RNA isolation and real-time PCR analysis

Total RNA was extracted using the HP Total RNA Isolation Kit (OMEGA) in accordance with the manufacturer’s standard protocol. First-strand cDNA was generated using a random primer and Moloney murine leukemia virus reverse transcriptase (Promega). Real-time PCR was conducted with SYBR Green PCR Master Mix (Bio-Rad) on a CFX Connect Real-Time System (Bio-Rad). The specific primer sequences were as follows: 5′-GAGCATCAAGATAAGATCAAAGCA-3′ (forward) and 5′-CTTCACCTTTCTCTTTGAATGGTT-3′ (reverse) for Renila, 5′-CAACTGCATAAGGCTATGAAGAGA-3′ (forward) and 5′ -ATTTGTATTCAGCCCATATCGTTT-3′ (reverse) for Luciferase.

### DNA affinity-binding assay

The DNA affinity-binding assay was performed, as described in previous studies with modifications^26^,^42^,^43^. Biotinylated oligonucleotides containing the κB DNA element sequence (5′-AGT TGA GGG GAC TTT CCC AGG C-3′) and the control oligonucleotide YY1 promoter sequence (5′-CGC TCC CCG GCC ATC TTG GCG GCT GGT-3′) were annealed with the corresponding antisense oligonucleotides. Nuclear extracts were isolated using a cytoplasmic/nuclear protein extraction kit as described above. A total of 6 μmol biotinylated DNA oligonucleotides, 100 μg control nuclear extracts and 30 μl of Streptavidin M280 magnetic beads (Invitrogen), which was washed three times with 400 μl binding buffer containing 20 mM Tris-HCl (pH 7.5), 75 mM KCl, 1 mM DTT and 5 mg/ml BSA were mixed in 500 μl binding buffer in the presence of 100 μg Salmon Sperm DNA (Invitrogen), and incubated for 2 h at 4 °C. The beads were collected and washed thrice with 500 μl of binding buffer. Subsequently, 100 μg of nuclear extracts was mixed with the beads in 500 μl of binding buffer with 13% glycerol and 100 μg of Salmon Sperm DNA overnight at 4 °C. The beads were collected and washed three times with 500 μl of binding buffer. The bound proteins were eluted by boiling in Laemmli sample buffer and then analyzed by immunoblotting.

## Additional Information

**How to cite this article**: Liu, Q. *et al.* Human Bocavirus NS1 and NS1-70 Proteins Inhibit TNF-α-Mediated Activation of NF-κB by targeting p65. *Sci. Rep.* **6**, 28481; doi: 10.1038/srep28481 (2016).
